# 

**Published:** 1817-06

**Authors:** 


### CATALOGUE OF MEDICAL BOOKS.

A Picture of the Present State of the Royal College of Physicians of London; containing Memoirs, Biographical, Critical, and Literary, of all the resident Members of that Society, and of the Heads of the Medical Boards, with some other distinguished professional Characters; to which is subjoined, an Appendix, containing an Account of the different Medical Institutions of the Metropolis, Scientific and Charitable, with their present Establishments. 16s.

A Fifth Edition of Mr. Forster's Observations on the Perennial Retreat of the Swallow.

Observations on the deranged Manifestations of the Mind, or Insanity; by J. G. Spurzheim, Licentiate of the College of Physicians in London, Physician to the Austrian Embassy, &c.

An Essay on the Shaking Palsy; by James Parkinson.

Commentaries on Scirrhus and Cancer; Part II. By William Thomas, Member of the College of Surgeons, and late Surgeon to the Forces.

An Essay on the Mode by which Constitutional Disease is produced from the Inoculation of Morbid Poisons. By Charles Salt, Member of the Royal College of Surgeons, London.

A Botanical Description of British Plants in the Midland Counties, particularly of those in the Neighbourhood of Alcester; with occasional Notes and Observations. To which is prefixed, a short Introduction to the Study of Botany, and to the Knowledge of the Principal Natural Orders. By T. Purton, Surgeon, Alcester. Embellished with 8 coloured Engravings; by James Sowerby, F. L. S. 2 vols. 20s.

Mr. Gray, of No. 27, Cross-street, Hatton-garden, will begin his Summer Course of Botanical Excursions into the Environs of London, with Practical Demonstrations of the Plants collected in them; on Tuesday the 3d of June, and continue them twice a week. —Mr. Gray has been induced to adopt this method of teaching in preference to the more formal method of lectures, as better adapted for the improvement of pupils.

Mr. Thomson will commence his Lectures on General and Medical Botany, on Tuesday the 3d of June, at two o'clock, in the Anatomical Theatre, Blenheim-street.

### A METEOROLOGICAL JOURNAL,

### NOTICES TO CORRESPONDENTS.

*We feel greatly obliged to* Senex *for his hints, and highly flattered by the compliment he pays our Reviews. To him, we need not plead the difficulty with which writers competent to such researches are procured, or stimulated to work. We shall, however, be thankful, if he will inform us of the books received and left so long unnoticed. To two, we plead guilty, but, in our defence, urge that they have been more than twenty months waiting for the judgment of an eminent surgeon. Our remedy must be, as in many other cases, to learn his opinion orally, or to send our own remarks for his correction.*

*We trust our present Number will show sufficient marks of industry, impartiality, and competency, in our various coadjutors. Our next, the first of our* Thirty-eighth Volume, *may, probably, be introduced with a Retrospect, in which we may notice several books and papers, omitted by accident or from necessity. To conclude, we trust the manner in which Senex's hints are attended to, will encourage him and others to remind us often of our duty, or to suggest further improvements in our plan.*

*We are much obliged to our* Doncaster Correspondent *for his interesting paper, but, without a better acquaintance, cannot insert a communication impeaching the credit of another Journal, and containing matter connected with so important a branch of pathology.*

*We thank* Chirurgo-Pharmacopolus *for his remarks, most of which he will see in our additional pages. He will also perceive, that we have taken care of his underscored words, and some others of the same kind shall be included in our extracts. Such is the only notice we ever take of grammatical errors, when the meaning is not obscured. Judges of correct composition will detect them, and to others they are unimportant.*

Juvenis *must excuse our once more postponing the hints we have collected relative to his reading. At present, we have only room to advise him to keep up, or to renew his classical learning, if he ever expects to appear in the higher branches of the profession,—an object every practitioner should have in view. We would likewise recommend him to read every day a portion of Celsus, and, if he retains any Greek, of Aretæus also. He should avail himself of the season to gain a little practical botany; but not unless he has some friend who can teach him the names of plants, whether in English or Latin. If he has no better opportunity of seeing the viscera, let him repair to a butcher, and learn their figure and situation in brutes, at the same time comparing them with Cheselden, Winslow, or any other anatomical writer. He should always have Henry or Duncan at hand, but never fail to consult the London Pharmacopœia in Latin. If there is no Latin copy in the shop, he should procure one.*

*The Editors wish to express their thanks to* Mr. P. *for his early answer.*

*For our next Number, we are promised Reviews of several important Chirurgical Works which have been too long delayed.*

*We feel particularly thankful to our* Warminster Correspondent, *and regret that his paper arrived too late for this Number.*

*Communications are received from* Dr. Kinglake, Messrs. J. Hoare, Bish, Powis Gitton, Ward, J. Isaac; Veritatis Curator, A. B. *&c.*

---

ERRATA.

Vol. 36, page 358, line 19, for *convex,* read *concave.*—Page 359, line 4, for *Parrey* read *Larrey.*

Vol. 37, page 353, line 3, for *Villeorne* read *Villermé.*—Same page, last line, for *joint* read *point.*—Page 354, line 2, for *coagulated* read *coagulable.*

---

*Our Correspondents will please to be particular in the Address of their Communications, which should be thus,*—"To the Editors of the London Medical and Physical Journal, at Mr. Souter's, No. 1, Paternoster-Row; or at Mr. Adlard's, 23, Bartholomew-Close."

### INDEX

### TO THE THIRTY-SEVENTH VOLUME.

ABDOMEN, caution to be observed after parturition 220
Abdominal viscera, transformation of the 157, 346
Abernethy, Mr. on his method of giving mercury 335
————, on the dietetic regimen in chronic diseases 406
Absorbents, on the functions of the 517
Absorption, on the powers of 60
Abstinence, considered as a moral duty 246
Accouchement, observations on remarkable cases of 194
———— among the Arabs, described 257
Acids, their effect on the animal fibre 68
Act, on the new surgeon's 146
——, observations on the apothecaries' 490
Adams, Dr. his Life of Hunter announced 172
————, his correction of an error in that work 350
————, Sir William, on the restoration of vision, 211; notice of his enquiry into the failure of operations for the cataract 350
Adder, one found in a block of coal 431
Adhesion, on the principles of 515
Affusion, observations on cold 311
Albers, Dr. on a change of colour produced by the nitrate of silver 152
Alcohol, a substitute for it in anatomical preparations 180
Aldis, Charles, his remarks on cancer 270
Air, on the purification of the 167
Alkalies, their efficacy in dyspepsia 271
————, their effects on the animal fibre 68
Allinhofer, Dr. on the nature and treatment of syphilis 36
Amaurosis, observations on 392
Amblyopy, remarkable case of 280
America, on the yellow fever of North 39
Amputation, case of, at the instep 354
————, improvement of silk ligatures for 520
ANALYSIS of new publications 51, 127, 220, 305, 394, 488
Anatomy, the ordinary divisions of it rejected 169
————, observations in pathological 170
————, substitute for alcohol in preparations of 180
————, review of a practical essay on pathological 519
Anchylosis, a cure for caries of the spine 158
Anchylosed joints, occasioned by scarlatina 277
Andrews, Mr. elected surgeon of the London Hospital 82
Anecdotes of Mr. Royston 114
———— of Dr. Menuret 206
————, singular one of G. F. Roulle 244
———— of Dr. Watson 379
Aneurism, case of that of the femoral artery 154
————, improved operation for 377
————, case of aortal 420
————, method of operating for external 500
————, inguinal, cured by tying the iliac artery 509
Animal fibre, on the cohesion of the 63
Animals, cruelty of experiments made on living 110
————, necessity of azote in the food of 343
Ankle, successful operation in a scrofulous 522
Antispasmodics, on their use in hooping cough 227
Aorta, case of aneurism of the 420
————, total obliteration of the 527
————, on the descending one of the dog 99
Apothecaries, address to the Society of 78
————, circumstances respecting the Act relating to 489
————, observations on the Act 490
Apprentices, on the hospital charges for 82
————, advice to medical 390
Aqueous affusion, observations on 311
———— membrane, on the issue of the 257

Arabs, practice of midwifery among the 257
——, singular adventure with the 475
Arm, case of diseased 239
Armstrong, Dr. his observations on fevers 305
Arsenic, case of supposed poison by 430
Artery, tumor removed from the face by tying the carotid 152
——, case of a wound in the peroneal 500
——, case of extraordinary disease of the humeral 508
——, inguinal hernia cured by tying the iliac 509
Arteries, why found empty in death 55
——, on the tonic power of the 56
——, on the vertebral 101
——, effects of the different methods of obliterating the 500
——, on the functions of the 517
Asthma, effects of a peculiar regimen in 394
Asylums, on the state of lunatic 409
Atmosphere, its influence on the animal system 167
Atkinson, Mr. explanatory statement concerning the paper of 91
Authors, on the complaints of 204
Automatic motion, remarks on 135
Azote, necessity of food containing 343
Bacot, John, his medical history of the first battalion of guards 325
Baines, Mr. on purifying unsound corn 80
Balfour, Dr. his reply to the reviewer of his Treatise on Rheumatism 199
——, vindication of his claim to priority on the reunion of separated parts 472
Balls, effect of some lost in the cavity of the thorax 29
Banks, Sir Joseph, illness of 156
Bark, effects of Peruvian 67
——, its effects on cynanche 68
——, improved mode of preparing 84
Barton, Dr. on the impurities of water 397
Bath, proceedings of the philosophical society at 426
Baths, cutaneous diseases cured by medicated 527
Battersea, botanical excursions at 340, 533
Beclard, Dr. his case of the transformation of the viscera 157
Beclard's, Dr. further observations on the same 346
Bedingfield, Mr. extracts from his selections 218
Beech, Archibald, on the gas produced by coal tar 329
Bell, John, observations on his case 261
——, his new treatise on practical surgery 351
——, announcement of his Consulting Surgeon 456
Bethlem Hospital, observations on the maniacs in 110
——, donations to 411
——, on that of Edinburgh 413
Bibliography, medical 168, 208
Bichat, his divisions of the animal frame 59
Bilious complaints, remarks on 143
Bill, observations on the surgeons' 146, 204
Binning, Lord, letter to him on lunatic asylums 409
Bitters, on those used in medicine 67
Bitumen, on the oil of 339
Bleeding, the death of Dr. Rush 46
——, observations on 12
——, on the use of it in severe injuries 97
——, beneficial in diabetes 104
——, on its utility in hooping cough 228
Blindness, cured by moxas 170
Blizard, Sir William, on the conduct of 82
Blunt, Mr. his Meteorological Register 87, 175
Blood, on the momentum of the 51
——, on the state of it in diabetes 105
——, defective determination of the 139
——, dangerous effects of extravasated 256
——, on the evolution of heat during the coagulation of the 387
——, experimental inquiry into the same 388
Blows, how to prevent ill effects from 341
Boggie, John, his case of gunshot wound 500
Bone, microscopic observations on the structure of the 325
——, on the condition of it in rickets 325
Books, on the choice of medical 113, 198
——, catalogue of medical 88, 176, 264, 352, 440, 534

### INDEX.

Books, new medical ones announced 88, 172, 263, 350, 351, 435, 436
Borden, on his theory of secretion 208
Bostock, Dr. his observations on diabetic urine 187
Botanical excursions round London 340, 425, 533
Botany, review of a treatise on practical 248
Brain, on the effects of extravasation on the 77
——, case of chronic inflammation of the 174
——, observations on the structure of the 290, 349
——, recent demonstration of the 427
——, examination of the structure of the 428
Brand, Mr. appointed secretary to the Royal Society 81
Bread, method of making good from unsound corn 80
———, improvement in the making of 160
Bres, M. his observations on caloric in the human body 283
Briggs, James, on an improved mode of operating for aneurism 377
Brodie, Mr. on the treatment of varicose veins of the legs 155
Bronchitis, on 221
Brookes, Mr. his account of Mr. Royston 114
————, his account of fossil bones 522
Buboes, observations on the matter of 37
Burrows, Dr. his remarks on the bill for regulating madhouses 263
————, his observations on the Apothecaries' Act 488
Buxton, Dr. his remarks on climate and consumption 360
————, reply to him on that subject 456
Cadet, M. on the honey of Mount Hymettus 85
Cadiz, on the fever at 237
Cæsarean operation, case of 194
————, account of it performed through the vagina 365
Cairo, account of the plague at 477
Caldwell, Dr. on contagion 42
Caloric, on the manner of its circulation in the system 283
Calomel recommended in dysentery 314
Cambridge, account of the fever at 484
Cancer, on the different methods of treating 191
———, observations on diseases confounded under that term 270
———, case successfully treated by pressure 354
———, effects of a peculiar regimen in 394
Capillary tubes, on the rise of fluids in 426
Carditis, observations on 324
Caries, observations on 158
———, caused by rheumatism 468
Carotid, delineation of the left one of a ram 100
———, wound in the face cured by tying the 152
———, tumour removed by the same operation ib.
Cartilages, ossification of those of the larynx 155
Cartmel, on the mineral waters of 267
Cases of—small-pox after vaccination, 2, 8; premature parturition artificially excited, 15; the successful use of colchicum, 17; uncommon disease in the head, 18; two of tetanus, 19, 21; of secondary small-pox, 27; gun-shot wounds, 31, 32; Professor Saussure, 70; Dr. Rush, 81; a peculiarity in the teeth, 89; bleeding in severe injuries, 97; diabetes mellitus, 106; Mr. Royston, 116; puerperal fever, 117; chorea sancti viti, 151; a change of colour in the skin, 152; reunited fracture, ib.; a wound in the face, ib.; a tumour successfully treated, ib.; aneurism of the femoral artery, 154; ossification of the larynx, 155; gunshot wound of the shoulder-joint, ib.; transformation of the viscera, 157, 346; the benefit of henbane in inflammation, 163; croup, 164; blindness cured by moxa, 170; hæmorrhage, 174; scarlatina, 177; diabetes, 186; the Cæsarean operation, 194; extra uterine pregnancy, 195; cataract, 213; the reproduction of the vitreous humour of the eye, 215; new method of operating for cataract, 216; peritonitis, 215; diseased arm, 239; vaccination, 255; typhus, 256; wounds in the cornea, 257; menstruation in old persons, 261; corpulency, 262; dyspepsia, 271; on one of inflammation, 272; an uncommon one proposed as a prize question, 275; on a wound of the eyebrow, 280; of tetanus, 320, 322; inflammation of the muscular structure of the heart, 323; tetanus, 329; imperfect vision, 331; puerperal fever, 327; hydrocephalus, 344; lameness cured by fracture, 345; amputation at the instep, 353; cancer, 355; the Cæsarean operation through the vagina, 365; the use of opium in tetanus, 371; an improved method of treating aneurism, 378; Dr. Rush, 385; atonic gout, 402; ardent fever, 414; enlargement of the heart, 416; pneumonia, 419; aneurism of the aorta, 420; idiotism, 428; a re-united finger, 432; a polypus in the stomach, 433; enteritis, 445; observations on that of Dr. Rush, 448; fever, 452, 485; a wound of the peroneal artery, 500; gunshot wound and fracture of the tibia, ib.; inguinal aneurism, 509; hernia of the dura mater, 510; an extra-uterine fœtus, ib.; scrofulous ankle, 522; fatal obliteration of the aorta, 527; obesity, 528.

Cataract, on removing that produced by the conical form of the cornea 211

———, new method of operating for the 216

———, observations on that method 273

———, on operations for the 455

———, Professor Scarpa's improvements in the treatment of 496

Caution to apothecaries in mixing medicines 432

Cavern, effects of mephitic vapour in a 474

Celibacy, physicians formerly bound to observe 86

Celsus, his observations on the methodic sect 235

Cerebellum, observations on the 349

Chalk, lizard found in a bed of 162

Chancres, on the poison of 35

Chapman, Edward, his observations on the new method of operating for cataract 273

Charlton, Kent, botanical excursions at 533

Charnock, Robert, on the mineneral waters of Cartmel 267

Chaumeton, Dr. on legal and judicial chemistry 165

———, extracts from his medical bibliography 168, 208

Chemistry, on legal and judicial 165

———, observations in 209

———, explosion produced by a preparation in 432

Chichester, Dr. introduced vaccine inoculation in America 339

Children, on the diseases of 351

China, method of preparing mercury in 525

Chirurgical operations, practical view of 156

Chirurgicus, answer to him on the depletory practice 12

Chisholm, Dr. his opinions on contagion controverted 42

Chorea sancti viti, case of 151

Cinnamon, etymology of the word 522

Circulation, heat produced by increased 53

Civilization, effects of 167

Clarke, Bracy, on the gripes of horses 145

———, W. S. his answer to Chirurgicus on depletion 12

Climate, its effects on consumption 360

———, that opinion invalidated 456

Cloquert, M. his anatomical observations 169

Clutterbuck, Dr. on the fevers of the metropolis 523

Coagulation of the blood, on the heat evolved by 387, 388

Coal, a snake found in a rock of 431

Cobalt, its existence in meteoric stones 343

Cohesion, on that of the animal fibre 63

Colchicum, successful in hysteria and hypochondriasis 17

Cold affusion, observations on 311

Coldefy, M. on new pharmaceutical preparations 83

Coleridge, Rev. Mr. his secondary case of small-pox 27

COLLECTANEA MEDICA 36, 114, 206, 290, 379, 474

Collier, Charles, his case of wound in the face 152

———, case of aneurism 154

Colour, that of the skin changed by nitrate of silver 152

———, on that of the skin and hair 209

———, on the incapacity to distinguish the difference of 331

Combustion, on human 167

Compositions, comparative quantities in pharmaceutical 191

Comrie, Peter, his reports on the ardent fever of the West Indies 413
Confectioners, dangerous practices of 165
Congestive typhus, observations on 315
Consumption, on its frequency at Malta 96
————, on a species common in England 331
————, effects of climate on 360
————, effects of a peculiar regimen in 395
————, that opinion corroborated 456
Contagion, French report on 38
Contractions, on those which succeed ulceration of the skin 510
Cooke, William, on a substitute for alcohol 180
Cookery, account of a remarkable work on 172
Cooper, Astley, his experiments on dogs 99
Copenhagen, proceedings of the medical society at 258
Copper, danger of the preparations of 166
Corn, method of using unsound, in bread 80
——, process for sweetening musty 156
Cornea, on its assumption of a conical form 211
——, on puncturing the 216
——, on wounds in the 257
Corpulence, remarks on 245
————, singular case in a child 262
————, two enormous instances of 528
Correspondents, notices to 88, 176, 264, 352, 440, 536
Corrosive sublimate, remarkable effects of 157
Coutanceau, M. his chemical observations 209
Cow-pox, cases of small-pox after 2
————, state of it in France 158
————, its preventive power 255
————, on the conveyance of the matter of it to America 374
————, on the antiquity of it 531
Cows inoculated with variolous matter 526
Crabbe, the Rev. Mr. his description of a poor house 109
Crampton, Philip, his method of operating for aneurism 500
Craniology, progress of that system 160
Craniology, explanation of 290
————, vindicated 297
————, on the unfavourable reception of 348, 526
Crauford, Dr. his enquiry into the effects of tonics 63
Critical Review, animadversions on the 93
Cross, Dr. his account of two cases of tetanus 19
——, Mr. on the improvement of silk ligatures in amputation 520
Croup, successfully treated with henbane 164
Cruveilheur, Dr. account of the pathological anatomy of 170, 519
Currie, Dr. simplified too much on the aqueous affusion 311
Curtis, Mr. appointed surgeon to the Dispensary for the Ear 171
————, his manufacture of artificial ears 253
Cutaneous diseases, efficacy of sulphurous fumigations in 85
————, on the arrangement of 419
————, on the method of curing them by nitro-muriatic baths 527
Cynanche maligna, effect of bark in 68
Cyprus, account of the plague in 481
Davy, Sir Humphry, on the virtue of magnesia 429
——, Dr. John, on the evolution of heat in the coagulation of blood 388
——, Edmund, his improved method of making bread 160
Deaf persons, invention of ears for 253
————, remarks on the disorders of 258
Death, on the appearance of the vessels in 55
Decandolle, M. his opinion on rye 162
Deglutition, case of impeded 155
Delivery, case of premature 15
————, on difficult cases of 91
————, unfortunate case of 219
————, case performed by a section of the uterus from the vagina 365
Demerara, description of the climate of 317
Denmark, state of vaccination in 258
Density, on that of water 525
Dentes sapientiæ, on the extraction of the 443
Depletion, on the effects of general 12
————, improper in typhus 316

Depletion, beneficial in diabetes 107
Desgenettes, M. his opinion on the yellow fever 41
Dewar, Dr. on the preservation of lime water 419
———, on the treatment of sinuous ulcers 513
Diabetes, facts and observations on 186
———, on the urine in 189
Diagnosis, contributions to 240
Diatrition, an ancient dietetic custom 246
Dickson, Dr. on the tropical endemic or yellow fever 233
——— observations on tetanus 326
Dieby, Dr. his remarkable cases of corpulency 528
Diet, proper one for corpulent persons 246
———, benefit of an aqueous 394
Disease, description of an uncommon one of the head 18
———, on that of Dr. Rush 448
———, on constitutional 517
———, extraordinary one of the humeral artery 508
Diseases, on those reputed contagious 50
———, on the common origin of 137
———, reports of 174, 263, 351, 436, 532
———, on mental 241
———, on those of dumb persons 258
———, peculiar regimen in certain 394
———, on the arrangement of cutaneous 419
———, new method of curing cutaneous 527
Dispensary, French translation of Garth's 433
Dissections 73, 116, 120, 157, 196, 197, 219, 223, 323, 330, 417, 422, 428, 446, 470, 505, 510
Dogs, effect of chancre poison on 36
———, on the inhumanity of subjecting them to experiments 168
———, on the proper food of 343
———, experiments on 521
Domeir, Dr. on the climate of Malta 361
Donnall, R. S. his trial for murder 429
Draper, R. L. on some remedies used in fevers 240
Drink, caution in regard to 247
Droitwich, description of a well-regulated mad-house at 109
Dropsy, on the effusions in 129
———, pathological observations on 128
Dubuisson, Dr. on insanity 241
Dumeril, Prof. his divisions of anatomy 169
———, his anniversary oration to the faculty of Paris 259
Dumourier, anecdote of 207
Dunn, Mr. on a case of scrofulous ankle 522
Dunning, G. T. his case of cancer treated by pressure 354
Dura mater, case of hernia of the 510
Dysentery, benefit of calomel and opium in 314
———, treatment of it in mild cases 532
Dyspepsia, case of, successfully treated with alkalies 271
———, observations on consumptive 334
Ear, new instruments for the 81
———, dispensary instituted for diseases of the 171
———, invention of an artificial 253
Earle's, Henry, case of hernia of the dura mater 510
———, on ulceration of the skin ib.
Edinburgh Medical Journal, review of the 232, 413
——— Review, on the conduct of the 297
———, account of the lunatic asylum at 411
———, treatment of Spurzheim at 526
Edwards, Dr. on his evidence in a late trial 430
Effusion, on dropsical 128
Egypt, particulars observed in a tour through 474
———, on the plague in 477
Embden, Dr. on the syphilitic poison 36
———, on the use of henbane in inflammation 163
———, on the treatment of hooping cough 220
Emetics beneficial in hooping cough 228
——— useful in typhus 310
Endemic, on the causes of the tropical 233
Enteritis, case arising from constipation 263
———, fatal case of 445
Epidemics, on the contagiousness of 41
Epilepsy, an hereditary disease 137
———, lapis infernalis given internally in 166
———, particular one caused by an affection of the spine 262
Errata, notices of 176, 350, 352, 536
Erysipelas, effect of bark in 68

Erysipelas, antiphlogistic regimen necessary in 314
Evolution of heat, observations on 388
Excursions, account of botanical 340, 425, 533
Exercises used by the ancients to remove disease 246
Experiments on the animal fibre 65, 67
———, on those of Dr. Parry 100
———, inhumanity of those made on living animals 110, 168
——— for sweetening musty corn 156
——— on bread 161
——— on the mineral waters of Cartmel 267
———, method of conducting 270
——— on animal heat 283
——— to ascertain the qualities of different nutriment 343
——— on the solution of salts 382
——— on the evolution of heat in wood 388
——— on damaged flour 429
——— on the venom of the viper 434
——— on obliterating the arteries 500
——— on the effect of ligatures in amputation of the thigh 521
——— on fossil bones 522
——— on flame 524
——— on garden mould and decayed stone 525
——— on inoculating cows with variolous matter 526
——— on intestinal gas 529
Explosions produced in chemical operations 432
Extravasation, on that of the brain 77
———, dangerous effects of 256
Eyes, account of new instruments for the 81
——, case in which they were frozen 170
——, successful operation on the ib.
——, on the restoration of sight to the 211
——, reproduction of the vitreous humour of the 215, 455
——, new mode of operating for cataract in the 216
——, on wounds in the 257
——, observations on the new mode of treating disorders of the 273
——, on a wound of the right eye-brow 280
——, connexion between the 282
——, case of imperfection in the 331
——, on diseases of the 392
——, observations on the operation for artificial pupil of the 496
Face, wound in the, cured by tying the common carotid 152
——, tumor successfully removed from the ib.
Fallopian tube, fœtus found in the 510
Fat, on the origin of human 507
Females, disproportion of mortality among 174
———, ruptures in 253
Femoral artery, case of aneurism of the 154
Fever, on the contagiousness of the yellow 38
——, case of puerperal, in which the uterus and spleen were chiefly affected 117
——, observations on the causes of the yellow 233
——, on that of Cadiz 207
——, case of pneumonia with 239
——, on some remedies used in 240
——, blue nose not a fatal symptom in typhus 256
——, practical illustrations of typhus and other 305
——, observations on the term 306
——, on the open forms of 309
——, emetics and purgatives recommended in 310
——, on the treatment of inflammatory typhus 312
——, treatment of congestive 315
——, cautions against depletion in 316
——, queries in a case of puerperal 337
——, reports on the ardent one of the West Indies 413
——, prevalence of typhus 436
——, account of one at Ipswich 451
——, observations on that which occurred in 1815 at Cambridge 484
——, London remarkably free from 523
Fibre, effects of tonics on the animal 63
Finger, case of one reunited after being cut off 432
———, observations on the cure 472
Flame, experimental observations on 524
Flookburgh, on the mineral waters of 267
Fluids, on their rise in capillary tubes 426
Flux, menstrual, at an advanced age 261
Fœtus, structure of a monstrous 151
———, effects of the venereal disease on the 335
———, account of an extra-uterine 510

Fontana, his observations on the poison of vipers 435
Food, necessity of its containing azote 343
Formulæ, on those of ancient physicians 342
Fossil bones, account of some extraordinary 522
Fox, Mr. on extracting the dens sapientiæ 443
Fracture, case of the os humeri 152
———, lameness cured by one 345
——— of the tibia, case of 500
Frost, its effect on the eyes 170
Fumigations, sulphurous ones efficacious in cutaneous disorders 85
Gales, Dr. on sulphurous fumigations ib.
Gall, Dr. on the craniological doctrine of 135
———, estimate of his opinions 290
Galls, their effects on the intestines 67
Gardanne, Dr. his advice to women 520
Garth's Dispensary, French translation of 433
Gas, coal tar or bitumen recommended to be used as 339
——, analysis of intestinal 529
——, benefit of baths of 527
Gibbon, James, his case of premature parturition 15
Gibney, Dr. his case of pneumonia 419
Gibraltar, on the mortality at 465
Ginger, beneficial in rheumatic gout 342
Goodlad, William, his case of tumor in the face 152
Gordon, Dr. his controversy with Dr. Spurzheim 160, 290
———, his attack on Mr. Abernethy 297
———, on the heat evolved in the coagulation of the blood 388
Gout, ginger recommended in rheumatic 342
Greenstone, experiments on decayed 525
Griffith, Dr. on the cause and prevention of hepatitis 143
Grimme, M. his account of a Cæsarean operation 365
Gripes, observations on the 145
Guards, medical observations on the first regiment of 325
Gun-shot wounds, cases of 29
——— of the shoulder-joint 155
———, case of, in the tibia 500
Guthrie, G. J. his case of wound of the peroneal artery ib.
Gymnasium, used to remove disease 246
Hæmorrhage, case of 174
——— from the uterus or vagina 532
Hair, its colour affected by the sun 209
Hall, Dr. Marshall, his contributions to diagnosis 240
Haller, on the Bibliotheca Anatomica of 169
Halliday, Dr. on the state of lunatic asylums 409
Hamilton, Dr. W. his account of a fever at Ipswich 451
Hampstead, botanical excursions at 425, 533
Harles, Dr. on a particular kind of apoplexy 262
Harrison, Thomas, on small-pox after vaccination 2
Harris, Mr. his meteorological reports 437, 439, 535
Haslam's Treatise on Insanity, remarks on 81
Hatchett, Mr. his process for sweetening corn 156
Haviland, Professor, his account of a fever at Cambridge 484
Head, on an uncommon disease of the 18
——, treatment recommended in that case 272
Health, on the means of preserving 210
———, mild seasons conducive to 263
Heart, on the force of the 55
———, inflammation of the 323
———, case of enlargement of the 416
———, on rheumatism of the 466
Heat, observations on animal 283
——, on its evolution in coagulation of the blood 387, 388
Heberden, Dr. on seasons conducive to health 263
Henbane, its virtue in inflammation 163, 164
Henning, Dr. on scarlatina 177
Hepatitis, on the cause and prevention of 143
Hernia, case of, in the duramater 510
———, number of male and female patients in 252
Herodicus, his use of exercise in the cure of diseases 246, 247
Hey, Wm. his reply to Mr. Atkinson 91
———, on the instruments invented by 265
———, effects of venereal disease on the fœtus 335
Hill, Mr. vindication of his Treatise on Insanity 81
Hippocrates, character of 259
Home, Sir Everard, on the origin of fat 507

### INDEX.

Home, Sir Everard, his analysis of fossil bones 522
Honey, on that of Mount Hymettus 85
Hooping-cough, on the nature and treatment of the 220
Horses, on the gripes in 145
Hospitals, on taking apprentices at 82
———, on purifying the air in 167
Howship, John, on the structure of bone 325
Hufeland, Dr. on the use of henbane in inflammatory diseases 164
Humeral artery, extraordinary disease of the 508
Humerus, case of fracture of the 152
———, case of gunshot wound where parts of it were removed 155
———, decay and transposition of the 470
Hungary, remarkable tempest in 165
Hunter, John, error in the life of 350
———, vindication of a passage in 502
———, objections to his operation in aneurism 504
———, on the principles of adhesion 515
Husson, Dr. on a polypus of the stomach 433
Hydrencephalus, observations on 93
Hydrocephalus, on an extraordinary chronic 344
———, frequency of the acute 351
———, case of, in a sheep 431
———, case of hernia connected with 510
Hydrophobia, prize questions on 83
Hymettus, on the honey of Mount 85
Hypochondriasis, success of colchicum in 17
———, observations on compound 243
Hyoscyamus, its use in hooping-cough 281
Hysteria, benefit of colchicum in 17
———, case of 26
Iliac artery, operation of tying the 154, 509
India, observations on bilious complaints in 144
Indolence, remarks on its effects in hot climates ib.
Infection, variety of scarlatina from the same 177
Inguinal aneurism, case of 509
Injuries, on bleeding in severe 97
Inflammation, efficacy of henbane in 163
——— of the brain 174
——— of the heart 323
Inflammatory typhus, observations on 312
———, prevalence of 532
Instruments, new ones for the eye and ear 81
Intelligence, Medical and Philosophical 78, 156, 251, 337, 424, 521
Inoculation, how constitutional disease is produced by 517
——— of cows with variolous matter 526
Insanity, vindication of a treatise on 81
———, on asylums for 409
Instep, case of amputation at the (with an engraving) 353
Instruments, observations on old surgical 265
Intestinal gas, observations on 527
Ipecacuanha, preparation of a tincture of 83
———, sugar of 84
Ipswich, account of a fever at 451
Iris, issue of the aqueous membrane of the 257
Iron, cobalt found in meteoric 343
Irritability, on that of the muscles 255
Jackson, Dr. on cold affusion in fever 311
Jamaica, on the healthiness of 363
Jenner, Dr. vindication of 532
Joints, observations on anchylosed 277
———, on the cause of caries in the 469
Kennedy, Alexander, his case of diseased arm 239
Kerr, George, his remarks on Dr. Parry 99
Kinglake, Dr. answers to 1, 114
Klaproth, death and character of 343
Klein, Dr. on chirurgical operations 156
Knife, new one for operations on the eye 82
Knowles, G. B. on the reproduction of the vitreous humour of the eye 455
Kraft, Dr. on the blue colour of the nose 256
Kretschmar, Dr. his method of curing cutaneous diseases 527
Labour, observations on difficult 91
Lambe, Dr. his reports on a peculiar regimen 394
Lameness cured by fracture 345
Lamercier, Dr. on a wound of the right eye-brow 280
Lancet, advantage of employing it 13
Langstaff, George, his account of an extra-uterine fœtus 510
Lapis infernalis given internally 166

4 A

Larrey, Baron, on gun-shot wounds in the thorax 29
———, on a case of paralysis 157
———, on caries of the spine 158
———, his cure of blindness 170
———, on wounds of the cornea 257
———, remarks on his paper concerning the reproduction of the vitreous humour 455
———, on rheumatism 466
———, on the cause of caries in the joints 469
Larynx, bony growth of the cartilages of the 155
Laurus cinnamomum, account of the 528
LECTURES announced—Drs. Latham and Southey on the practice of physic, 86; Dr. Adams, ib.; Mr. Taunton's on anatomy and surgery, ib.; Mr. Clarke on midwifery, ib.; Dr. Clough on the same, ib.; Dr. Adams, 172; Drs. Merriman and Ley, ib.; Dr. Davis on Midwifery, ib.; Mr. Curtis on the ear, ib.; Mr. Stevenson on the eye and ear, ib.; Mr. Clarke's, 263; account of Dr. Spurzheim's, 348, 427; Drs. Merriman and Ley, 350; Mr. Curtis, ib.; extract from the clinical of Dr. Pearson, 420; Dr. Davis on the theory and practice of midwifery, 436; Dr. Clutterbuck on the theory and practice of physic, ib.; Mr. Taunton on surgery, ib.; Mr. Gray's botanical, 535; Mr. Thomson's ditto, ib.
Leeches preferred to the lancet 12
Legh, Thomas, account of his journey in Egypt 474
Legs, on the treatment of varicose veins of the 155
Le Maire, Mons. on a peculiarity in the structure of the teeth 89
———, remarks on his paper 441
Leslie, Prof. his experiments on garden mould 525
Lettsom, Dr. account of his first acquaintance with Dr. Rush 46
———, not the first who sent vaccine lymph to America 339, 374
———, letter of Dr. Mease to 384
Ley, Dr., his case of puerperal fever 117
———, observations on the case 337
Ligature, on the application of it in aneurism 378
Ligature, wound of the peroneal artery successfully treated by 500
———, improvement in the silk 521
Lime-water, on the preservation of 419
Liver, observations on disorders of the 143
Liverpool, optical phenomenon observed at 350
Lizards found in a chalk rock 162
Lobel, Dr. on a new operation for cataract 216
———, remarks on his method 273
Lobstein, Dr. his account of a Cæsarean operation 194
———, case of extra-uterine pregnancy 195
London Hospital, proceedings of the election of a surgeon at the 82
——— Medical Society, proceedings of the 81, 522
———, on the prevalence of fevers in 523
Lucas, Mr. his case of pneumonia 239
Lunatic asylums, remarks on the state of 409
Madeira, on the climate of 364
Mad-house, specimen of a well-regulated 109
Madness, observations on 81
——— connected with delirium 243
M'Grigor, Sir James, on his medical statements 461
Magnesia vitriolata, its effect in strengthening the intestines 68
———, its efficacy in purifying musty flour 160, 429
Malconformations, on primitive 347
Malta, on the frequency of consumption at 96
———, on the mildness of its climate 361
———, reports of mortality at 457
Mangili, Professor, on the venom of the viper 434
Mantell, Gideon, his case of enteritis 445
Marcus, Dr. on hooping-cough 220
Marcet, Dr. on the medicinal properties of stramonium 337
Marshall, Mr. on the laurus cinnamomum 528
Mary-le-bone, magnitude of the parish of 174
Maunoir, M. his account of a monstrous fœtus 151
———, his letter to Prof. Scarpa on artificial vision 496
Mease, Dr. his account of the death of Dr. Rush 81

Mease, Dr., his letter to Dr. Lettsom on the same subject 384
Measure, necessity of the minim 14
———, observations on the same 103
———, use of it defended 112
———, remarks on an irregularity in 251
Medical bibliography 168, 208
——— Benevolent Society, anniversary meeting of the 523
——— and Philosophical Intelligence 78, 156, 251, 337, 424, 521
——— books, on the choice of 113, 198, 536
——— students, advice to 259
——— history of the first regiment of foot guards 325
——— Society of London, their meetings 81, 350, 522
Medicine, critical review of the state of 413
Medico-Chirurgical Transactions, review of the 70, 151, 323, 496
Mediterranean, on disorders in the 465
Membrane, on the issue of the aqueous 257
———, suture of that of the stomach 433
Menstruation, instances of it at an advanced period 261
Mental diseases, observations on 241
——— faculties, on the 135
Menuret, Dr. memoirs of 206
Mercury recommended in hepatic cases 143
———, Chinese method of preparing 525
Meteoric iron, cobalt found in 343
Meteorological journals 87, 175, 437, 438, 439, 535
Middlesex Hospital, lectures at 86
Minim, on the measure by the 14, 103, 112, 193, 251
Moisture, its influence on the irritability of the muscles 255
Montain, Dr. on a case of hydrocephalus 344
Montenegre, Dr. on the ancient formula of physicians 342
Morel, W. R. his case of gun-shot wound 155
Morrison, Dr. his treatise on tetanus 317
Mortality, annual one for 1816 173
Morveau, Guyton, tribute of respect to 167
Mould, observations on dried garden 525
Mountains, new method of measuring the height of 522
Moxa, caries of the spine relieved by 158
———, blindness cured by 170
Munich, remarkable accident at 432
Murder, remarkable trial for 429
Muriat of soda, its effects on the intestines 68
——— a substitute for alcohol 180, 426
Muscles, influence of moisture on the irritability of the 255
Mystic forms of ancient prescriptions 342
Nails, observations on blows of the 341
Nasse, Prof. on the irritability of the muscles 255
Neck, tumour removed from the 152
Nervous system, observations on the 134
Newcastle, remarkable disorder in the family of the Duke of 435
Newmarket, on the exercise practised there 247
Nicholl, Dr. his case of imperfect vision 331
Nicholles, John, on peculiar structure of the teeth 441
———, on extracting dentes sapientiæ 443
Nitrate of silver, its effect on cohesion 70
——— changes the colour of the skin 152
Nose, its blueness not a fatal symptom in typhus 256
Nourishment of animals, on the proper food for 343
Nux vomica, its efficacy in paralysis 157
Obesity considered as a disease 245
———, remarkable instances of 262, 528
Odier, Dr. his account of the illness and death of Prof. Scarpa 70
Ophthalmia produced by an affection of the head 19
Opium, proper mode of administering 67
——— improper in hooping cough 231
——— useful in dysentery 314
———, benefit of large doses of it in tetanus 322, 371
Oration, abstract of that delivered before the Medical Society 391
Organic productions, observations on 519
Ossification of the cartilages of the larynx 153
———, on the mechanism of 209

Pancras, magnitude of the parish of St. 174
——, report of the Small-pox Hospital at 254
Paralysis, on its cure by nux vomica 157, 345
Paris, proceedings of the vaccine committee at 158
——, public sitting of the faculty of medicine at 157, 259
——, patients admitted into the hospitals at 341, 529, 530
——, reigning maladies at 530
Parry, Dr. an account of his treatise on Pathology and Therapeutics 51, 127
——, remarks on his opinions 99
Parturition, case of premature 15
——, observations on difficult 91
——, caution to be observed after 220
Pathological anatomy, observations on 170, 519
Pathology, elements of 51, 127
Pearson, Dr. George, his case of aortal aneurism 420
Peritonitis, case of acute 218
Peroneal artery, case of wound of the 500
Peruvian bark, its effects on the system 67
——, new preparations of 84
Pettigrew, Mr. observations on his Life of Lettsom 339
——, his correction of an erroneous statement 374
Pharmaceutic preparations, account of new ones 83
——, table for ascertaining the properties of 192
Philadelphia, return of deaths for 363
Philips, Dr. on a species of pulmonary consumption 331
Phthisis, aortal aneurism connected with pulmonary 420
Physicians formerly compelled to celibacy 86
——, mystical forms of prescription used by the ancient 342
Plague, miscellaneous hints on the 477
——, observations on it in Egypt 478
——, account of it at Cyprus 481
Plants, catalogue of them round London 425, 533
Plethora, remarkable case of 351
Pleurisy, the death of Dr. Rush caused by 81
Plymouth, fossil bones found at 522
Pneumonia, case of with bilious fever 239
——, on blood-letting in 419
Poison, on the syphilitic 36
——, on the action of 209
——, how used by savages 210
Polypi, case of enlargement of the heart with 416
——, one found in the stomach 433
Polysarchia, remarkable case of 262
Poor-house, description of a 109
Practice, contrariety in surgical 191
Pregnancy, case of extra-uterine 195
Pressure, on the treatment of cancer by 354
Prize questions on hydrophobia 83
—— on a remarkable complaint 275
Prizes distributed at Paris 159
Puerperal fever, case in which the uterus and spleen were principally affected 117
——, queries on the case 337
Pulse, on the arterial 100
Pupil, on the operation for artificial 496
Quarterly Review, observations on the 81, 298
Questions, prize ones on hydrophobia 83
——, another on a remarkable complaint 275
Rabies contagiosa, distinction between tetanus and 321
Rachalgie, the caries of the spine so called 158
Ram, dissection of the carotid of a 100
Raven, Thomas, on the use of colchicum in hysteria and hypochondriasis 17
Read, Thomas, his cases of tetanus relieved by opium 372
Redhead, Mr. his cases of small-pox after vaccination 3
——, remarks on the cases of 8
Reeves, Dr. on the influence of air and water 397
Regimen, effects of a peculiar one in chronic diseases 394
Remedies, remarks on some used in fever 240
Remer, Dr. noble sentiment of 166
——, on effluvia in the atmosphere 167
——, on experiments made on living animals 168
Re-union, case of it after a separation of the parts 432
——, observations on 473
Review, answer to an uncandid 93

Review, reply to that of Dr. Balfour on rheumatism 199
——, answers to the Edinburgh 290
——, on the Quarterly 81, 298
Rheumatism, Dr. Balfour's defence of his treatise on 199
——, benefit of venesection in 314
——, on that of the heart 324
——, benefit of ginger in 342
——, on the metastasis of 466
——, cause of caries in the joints 469
Rhinoceros found in a fossil state 522
Rhubarb, new method of treating that of Moscow 84
Rickets, on the state of the bones in 325
Ring, Mr. his answer to Dr. Kinglake 1
——, his explanation on the same 114
Romans, punishment inflicted by them on negligent drivers 210
Roux, M. his memoir on the cure of paralysis 157
Royal Society, secretary appointed to the 81
——, resumption of its meetings 156
——, proceedings of the 522
Royston, William, memoirs of 114
——, appearances on the dissection of 116
——, reflections on his case 117
——, Mr. Stevenson delivers the anniversary oration instead of 261, 391
Ruptured Poor, report of the Society for the Relief of the 252
Rush, Dr. recollections of 46
——, particulars of the death of 48, 81
——, on the conduct of 338
——, anecdotes of his illness 384
——, remarks on his case 448
Rye, the spur of it not a champignon 162
Sabatier, M. on the curvature of the spine 346
Salisbury, William, account of his practical botany 248
——, his botanical excursions 340, 425, 533
Salmon, William, his description of instruments for the head 266
Salt, Charles, on constitutional diseases communicated by inoculation 517
Salts, effects of Glauber's 69
Salts, common, recommended for anatomical preparations 180
Saussure, Prof. account of his illness and death 70
Saws, description of old surgical 265
Scarificator, description of a new 81
Scarlatina, varieties arising from the same source of infection 177
——, anchylosed joints occasioned by 276
Scarpa, Prof. his improved operation in aneurism 377
——, on the operation for artificial pnpil 496
Sciatica, benefit of stramonium in 337
Scotland, on the frequency of insanity in 409
Scrofula, common in the deaf and dumb 258
——, observations on 335
——, effects of aqueous regimen in 394
Sea water, method of rendering it potable 346
Secretion, on theories of 208
Senex, his advice on the choice of medical books 198
Seton, fracture of the os humeris treated with the 152
—— successfully employed in fracture of the tibia 500
Sheep, case of hydrocephalus in a 431
Shoulder, gun-shot wound in the 155
Sicily, on the climate of 361
Silver, effects of nitrate of, on cohesion 70
——, the skin discoloured by nitrate of 152
Skin, on a change of colour in 152
——, on the colour of the 209
——, contractions succeeding ulceration of the 510
Small-pox, cases of after vaccination 2
——, cases of natural 3
——, remarks on the cases 8
——, history of recurrence in the same person 27
——, report of the hospital for 254
——, remarkable case of 255
——, occurrence of after vaccination 436
Smerdon, C. W. on diabetes 104, 186
Snake, one found in a block of coal 431
Soda, effect of muriat of 68, 180, 426
—— in a state of carbonate in mucus 209
Soden, J. S. his case of inguinal aneurism 509

Spencer, Samuel, his account of surgical instruments 265
Spine, on a caries of the 158
——, epilepsy produced by disorder in the 261
——, on the curvature of the 346
Spleen affected in puerperal fever 117
Spurzheim, Dr. his contention with the Edinburgh Reviewer 160, 290
——, his demonstrations of the brain 348, 427
——, his conduct at Edinburgh 526
Stansfield, Josias, case of fracture, by 152
Stanley, Edward, his case of inflammation of the heart 323
——, on the bones in rickets 325
Starr, R. N. on bleeding in severe injuries 97
Steinbruch, Mr. on a remedy for the tooth-ach 262
Stevenson, Mr. on new instruments for the eye and ear 81
——, abstract of his anniversary oration 391
Stewart, James, his case of enlargement of the heart 416
Stomach, on the cravings of the 25
——, effects of poisonous substances on the 209
——, account of a polypus in the 433
——, suture of the membrane of the ib.
Stramonium, on the internal use of the 287
——, on the medicinal properties of 337
Strasburgh, observations and cases in the lying-in hospital at 194
Students, advice to medical 259
Suffolk, lizards found in a chalk rock in 162
Sulphurous fumigations beneficial in cutaneous complaints 85
Sun, effects of the rays of the 209
Surgery, on the bill for regulating the practice of 146
——, contrariety in the practice of 191
——, remarks on the bill for regulating 204
——, on old instruments for 265
——, instructions to apprentices in 390
Sutton, Dr. on the frequency of consumption in Malta 96
——, reply to him on climate and consumption 360
Sutton, Dr. observations by him on the influence of climate 456
Sweetmeats, caution in regard to 165
Sydenham, character of 48
Sym, Mr. on the temperature of water 525
Syphilis, on the nature and treatment of 36
Table of cases of small-pox 5
——, one of the quantities of pharmaceutical compositions 192
Tar, use of the coal for gas lights 339
Tartar, salt of, recommended as a remedy for unsound corn 80
Taunton, John, on the use of opium in tetanus 371
Teeth, peculiarity in the structure of the 89
——, observations on the same 441
——, on the extraction of 443
Tempest, violent one in Hungary 165
Tetanus, descriptive account of two cases of 19
——, review of a treatise on 317
——, on the idiopathic 318
——, distinction between rabies and 321
——, cases of 322
——, observations on 326
——, cases of 329
——, benefit of large doses of opium in 371
Therapeutics, treatise on the Elements of 51, 127
Thilenius, his observations on tooth-ache 262
Thoracic viscera, transformation of the 157
——, further particulars of the same 346
Thorax, on gun-shot wounds in the 29
——, wound in the 97
Tibia, case of fracture of the 500
Tic douleureux, observations on the 136
——, benefit of stramonium in 337
Tinea capitis, on the benefit of turpentine in 101
Tonics, their effects on the animal fibre 63
Tooth-ach, benefit of nitre in 262
Transformations of the viscera 157, 346
——, observations on 519
Travers, Dr. his case of ossified larynx 155
Trial, remarkable one for murder 429
Tribolet, Dr. on henbane in inflammation 163
Tropics, on the endemics of the 233
Truss Society, report of the London 252

Tubes, on the ascent of fluids in the capillary 426
Tumour, case of one removed from the face and neck 152
Turpentine, its efficacy in tinea capitis 102
Turtle, observations on the blood of the 388
Typhus, blueness of the nose not a fatal symptom in 256
——, practical illustrations of 305
——, treatment of the inflammatory 312
——, treatment of congestive 315
——, on the prevalence of 436
Ulceration of the skin, on the 510
Ulcers, on the treatment of sinuous 513
Urine, observations on that in diabetes 107, 187
Uterine pregnancy, case of extra 195
Uterus, case of puerperal fever in which it was principally affected 117
——, effects of the venereal disease on the fœtus in the 335
——, section of it through the vagina 365
——, hæmorrhage from the 532
Vaccination, occurrence of small-pox after 2
——, remarks on cases of 8
——, report of the Committee of at Paris 158
——, report of the Small-pox Hospital on 254
——, on the introduction of it in America 374
——, on the antiquity of 531
Vagina, Cæsarean operation performed through the 365
——, hæmorrhage from the 532
Valentin, M. on the yellow fever 43
Valetudinarian, his observations on surgical practice 191
Vapour, remarkable effects of a mephitic 474
Varicose veins, treatment of the 155
Variolous matter, cows inoculated with 526
Veins, treatment of varicose 155
Venereal disease, its effects on the fœtus 335
Vertebra, case of wound in the dorsal 98
——, observations on caries of the 158
Vice, effects of rheumatic 467
Viper, on the venom of the 434
Virey, M. on the spur of the rye 162
Viscera, transformation of the abdominal and thoracic 157, 346
Vision, on the restoration of 211
——, case of curious imperfection in 331
Vitreous humour of the eye, on the re-production of 215
——, observations on the same 455
Wachsel, J. C. his report on small-pox and vaccination 254
Wadd, William, his remarks on corpulency 245
Walker, Richard, reply to him 26
——, his pharmaceutical table 192
——, on the reply of Mr. Woodham 193
——, answer to 288
——, his close of the controversy 422
Ward, Henry, on the minim measure 103
——, answer to 112
——, his observations in reply 251
——, on the internal use of stramonium 287
Water, on the mineral one of Cartmel 267
——, that of the sea rendered potable 346
——, benefits of distilled 406
——, on the preservation of lime 419
——, on the density and temperature of 525
Waterhouse, Dr. his letters to Dr. Lettsom 375
Watson, Bishop, memoirs of 379
West Indies, on the ardent fever of the 413
Wilkinson, Dr. his account of lizards found in a chalk rock 162
——, on the rise of fluids in capillary tubes 426
Wollaston, Dr. resigns the office of secretary to the Royal Society 81
Women, menstrual flux observed in aged 261
——, advice to them at the critical period of life 520
Wood, Kinder, his case of chorea sancti viti 151
Woodham, Mr. his reply to Mr. Walker 26
——, answer to 193
——, his rejoinder 288
——, his final answer 425
Woollcombe, Dr. on a case of cancer 354
Wounds, cases of gun-shot 29
—— in the thorax 97
—— in the dorsal vertebræ 98

Wounds in the face 152
——— of the shoulder-joint 155
———, on those of the cornea 257
———, on one of the right eye-brow 280
———, case of gun-shot, with fracture 500
Wounds of the peroneal artery 500
Yeatman, J. C. his case of amputation at the instep 353
Yeats, Dr. on hydrocephalus 93
Yellow fever, on the contagion of 38
———, observations on the 283

### PLATES.

M. LEMAIRE's Peculiarities in the Teeth . . . Page 90
Mr. YEATMAN's Case of Amputation . . . . 353

